# Spirituality and religiosity contribute to ongoing COVID-19 vaccination rates: Comparing 195 regions around the world

**DOI:** 10.1016/j.jvacx.2022.100241

**Published:** 2022-11-16

**Authors:** Jason P. Martens, Bastiaan T. Rutjens

**Affiliations:** a Department of Psychology, Capilano University, North Vancouver, Canada; b Department of Psychology, University of Amsterdam, Amsterdam, the Netherlands

**Keywords:** Spirituality, Religiosity, COVID-19, Vaccinations, Skepticism

## Abstract

Vaccine hesitancy has taken global prominence with the rapid spread of COVID-19, but what factors are related to this considerable variation in vaccination rates globally? Three studies that encompass 195 unique regions from around the world found that the relative spirituality and religiosity of a region predict ongoing COVID-19 vaccination rates, such that those regions higher in spirituality and/or religiosity are regions with lower COVID-19 vaccination rates. In Study 1, data from 23 regions globally were obtained, and both spirituality and religiosity were negatively associated with vaccination rates. These effects held when applying two methods to account for vaccine supply issues. In Study 2, data from 144 regions globally were obtained, and once again religiosity negatively predicted COVID-19 vaccination rates. It remained a significant predictor of vaccination rates when controlling for GDP, population age, collectivism, general skepticism towards vaccinations, and previous inoculation history. In Study 3, data from all USA states and the District of Columbia were obtained, and religiosity and spirituality once again were negatively associated with COVID-19 vaccination rates. Effects held controlling for other factors. Across studies, spirituality and religiosity account for a large amount of the variance in vaccination rates. These results suggest that real-world behavior can be predicted by the relative spirituality and religiosity of a region.

## 1 Introduction

Skepticism towards science—that is, the unwarranted rejection of science—can have detrimental impacts on health. One manifestation of science skepticism, vaccine hesitancy (i.e., the delay or refusal to get vaccinated), has taken global prominence recently with the rapid spread of the novel coronavirus (SARS-CoV-2) which leads to coronavirus disease (COVID-19). Vaccinations have been shown to reduce the spread of COVID-19 and are considered an effective way of managing it [1]. Despite their effectiveness, vaccine hesitancy exists. What factors might be associated with vaccine hesitancy?

Religiosity (institutionalized belief) and spirituality (individualized and intuition-driven belief) seem to be significant candidates. In terms of religiosity, many religious leaders have been active in the ongoing COVID-19 global pandemic. For example, Greek clerics have been prominent in anti-vaccine protests in Greece. Although attending church has been linked with higher COVID-19 transmission [2], Greek clerics have suggested that going to church is protected, as “God does not allow you to be infected” [3]. Religious freedom has been implicated in legal efforts against measures design to protect people during the pandemic. For example, in Canada, legal challenges have been issued on restricting church services due to COVID-19, arguing that they partly violate their rights on freedom of religion [4], and similar challenges to vaccine mandates have occurred in the USA [5]. In addition, the relative religiosity of a region in the United States is associated with both higher mobility and lower declines in mobility for stay-at-home orders related to the pandemic, which can affect transmission rates [6]. In terms of spirituality, recent work has shown that vaccine skepticism among more-educated Dutch parents is rooted partially in an intuitive epistemology, with respondents in qualitative interviews emphasizing the central role of the individual in determining what truth is [7]. Indeed, whereas religiosity is primarily based on an institutionalized set of beliefs, a common definition of post-Christian spirituality is an individualized set of beliefs characterized by a strong reliance on subjective experience and intuitive epistemology [8], [9]. In other words, spirituality is more individualistic and subjective in nature. Although distinct, both religiosity and spirituality have been shown to be related to vaccine skepticism [9], [10], with spirituality, on the individual-level, being the better predictor [9], [11]. Taken together, religiosity and spirituality are likely to have negative effects on efforts to contain the COVID-19 pandemic, of which vaccine hesitancy is an important part. We revisit the link between religiosity, spirituality, and vaccinations further in the discussion.

Research on factors contributing to vaccine hesitancy often focuses on a single region—typically a country. For example, participants’ level of spirituality and science literacy predict vaccine skepticism in the Netherlands, with higher spirituality and lower science literacy both being associated with higher vaccine skepticism [9], while among Americans, higher religiosity among participants predicted vaccine skepticism [10]. In addition, qualitative data suggests religion is a factor in polio vaccination boycotts in northern Nigeria, with religious leaders playing a role in organizing boycotts [12]. More specifically to COVID-19 vaccinations, a recent study in the United States found that COVID-19 vaccine hesitancy is predicted by a number of variables, including lower education and income of participants [13]. In Canada, social media posts about COVID-19 vaccine hesitancy suggest that safety concerns and suspicion about the vaccine/pandemic are contributing factors [14]. In both Ireland and the UK, younger age, lower income, and higher religious beliefs are all associated with COVID-19 vaccine hesitancy [15]. And in Australia, COVID-19 vaccine hesitancy is associated with higher religiosity and populist views [16]. These studies are informative for the given region, but they do not assess broader cultural influences that occur on the region-level.

By including region-level predictors, a different level of analysis is assessed than the individual-level, which can offer novel insights. Although somewhat counterintuitive, individual-level predictors might not directly map onto region-level predictors (and vice-versa). This is often referred to as Simpson’s paradox or an ecological fallacy, and can be a common occurrence in psychological science [17]. For this reason, it is important to not only assess individual-level effects, but also region-level effects of vaccination rates across different parts of the world.

There have been a limited number of studies that have assessed vaccination attitudes across countries. One study across 24 countries found fairly consistent effects, one being that antivaccine attitudes were highest among those high in conspiracy thinking [18]. A more recent global study assessed science skepticism of various domains, including vaccine skepticism, across 24 countries [11]. The main predictors of spirituality and science literacy were across cultures the most consistent predictors of vaccine skepticism, while religiosity was a less reliable predictor but still present in many of the included countries.

However, it is not clear whether these predictors generalize to ongoing COVID-19 vaccination efforts. For example, vaccine skepticism was measured via the belief that vaccines cause autism [11], which is not directly related to hesitancy towards COVID-19 vaccinations (also see [19]). Indeed, psychological research suggests that attitudes best predict behavior when the attitudes closely correspond to the behavior [20]. It also is not clear whether results on surveys that were taken before the current COVID-19 pandemic will generalize to behavioral outcomes during the ongoing pandemic. Empirical work on behavioral measures is needed.

The current research aims to assess the role of spirituality and religiosity as region-level predictors on current COVID-19 vaccination rates around the world. Importantly, vaccine availability is likely to have an effect on vaccination rates [21] and is a global problem [22], so several attempts were made to control for vaccine availability. Vaccine availability was controlled for in three ways: applying a 20% threshold to vaccine rates (Study 1), including gross domestic product (GDP) as a predictor (Studies 2–3), and testing for effects at multiple dates (Studies 1–3).

Justification for the 20% threshold is that low vaccination rates might reflect a lack of availability rather than vaccine skepticism. The 20% threshold eliminates countries that essentially lack access, but also allows for countries that might have relatively high skepticism. The assumption underlying the threshold is that those with relatively higher vaccination rates are more likely to have a greater supply.

However, because including a threshold decreases the sample size, alternative methods for accounting for vaccine availability were also utilized. GDP captures the relative wealth of a region and can distinguish between richer and poorer regions. Vaccine availability is likely influenced by region wealth, such that richer countries in particular are likely to have greater access to vaccines than poorer countries [21]. In other words, countries that have larger GDPs are likely to have greater access. Thus, including GDP in analyses can help distinguish whether effects are due to accessibility of vaccines.

Finally, testing the effects on multiple dates assumes that earlier availability would be relatively sparse compared to more recent time periods. That is because vaccine production and availability should increase as countries are better able to organize and purchase the vaccines, which should decrease availability concerns so that other factors (e.g., spirituality and religiosity) can exert a larger influence. In other words, spirituality and religiosity should have a greater effect on recent vaccine rates than earlier ones, where supply issues were a greater concern.

In addition, we also included collectivism in our analyses. Simply put, those from collectivistic cultures (e.g., Japan, Ecuador) tend to view themselves as connected to others and prioritize collective goals over individual goals to a greater extent than those from individualistic cultures (e.g., United States, Australia; [23]). Getting vaccinated does not just offer protection for the individual who is vaccinated, but also provides protection for those they interact with by being less likely to spread COVID-19 [24], such as by providing herd or community immunity. For this reason, vaccination rates might be higher in collectivistic cultures. Consistent with this prediction, collectivism has been found to influence mask wearing during the current COVID-19 pandemic [25].

We also included measures of vaccination history and vaccine skepticism. A history of vaccinations might translate into a willingness to get vaccinated for COVID-19. Indeed, a systematic review of the literature found that among several countries, having previous vaccinations was associated with a greater intention to get vaccinated during a pandemic [26]. For skepticism, research suggests that skepticism about vaccines has been found to influence actual vaccinations among at risk groups, including the elderly [27] and nurses [28].

Lastly, because many regions offer vaccinations to older individuals first and age is risk factor for the virus [29], the relative age of a population might influence vaccination rates. For these reasons, we controlled for the age of the population in all of the reported analyses.

The primary hypotheses guiding this research were that (a) spirituality predicts vaccination rates such that higher spirituality is related to lower COVID-19 vaccination rates, and (b) religiosity predicts vaccination rates such that higher religiosity is related to lower COVID-19 vaccination rates. Studies 1 and 3 tested hypotheses (a) and (b), while Studies 2 tested hypothesis (b). Across studies, two different measures of spirituality were used and three different measures of religiosity.

## 2 Material and methods

### 2.1 Spirituality

In Study 1, mean spirituality ratings for each country were taken from a recently published global study [11], which asked participants two items about their spirituality: the extent to which they considered themselves a spiritual person and the extent to which others considered them a spiritual person (see original source for full details). In Study 3, spirituality ratings were based on the percentage of adults who reported feeling “spiritual peace and wellbeing” at least once a week in each USA state and the District of Columbia, taken from the Religious Landscape Study [30].

### 2.2 Religiosity

In Study 1, mean religiosity ratings for each country were taken from the same sources where spirituality data were obtained [11], which included two items: “Religion is the one thing that gives meaning to life in all its aspects”, and”God has been defined for once and for all and therefore is immutable” (see original source for full details). While in Study 2, religiosity was the percentage of those who named a religion in the Wellcome Global Monitor questionnaire [31]. In Study 3, religiosity ratings were based on the percentage of adults who reported that religion was very important in each USA state and the District of Columbia, taken from the Religious Landscape Study [30].

### 2.3 Region wealth

For Study 2, GDP per capita in current U.S. dollars was used as an indicator of the region wealth. The most recent data available from the World Bank [32] or the Organization for Economic Co-operation and Development [33] was used. For Study 3, GDP was obtained for each state and the District of Columbia from the Bureau of Economic Analysis of the U.S. Department of Commerce [34], which was converted to per capita to be equivalent with Study 2 based on population data from the 2020 U.S. Census [35].

### 2.4 Collectivism

For Study 2, collectivism scores were used from Hofstede’s country comparison index [36]. Because this index has higher scores representing individualism and lower scores representing collectivism, they were reverse scored so that higher numbers represent collectivism. For Study 3, collectivism scores for each of the 50 U.S. states were used [37]. No collectivism score was available for the District of Columbia, so this region was not included when collectivism was included in the regression models. Both indexes [36], [37] were found to predict mask wearing during current COVID pandemic [25].

### 2.5 Vaccination history and skepticism

For Study 2, vaccination history and skepticism were values taken from the Wellcome Global Monitor questionnaire [31]. Vaccination history was the percent of respondents that had their children receive a vaccine from a childhood disease (e.g., polio, measles). The disease varied to be ones relevant to the country. Vaccine skepticism was the mean of three questions: “Vaccines are important for children to have,” “Vaccines are safe,” and “Vaccines are effective.” All responses were on a 5-point scale from strongly agree to strongly disagree. Reliability of the 3-item scale was good (α = 0.91).

### 2.6 Population age

The median population for each region in Studies 1–2 was obtained from an online database that uses official sources [38]. For Study 3, median age came from the U.S. Census [39].

### 2.7 COVID-19 vaccination rates

COVID-19 vaccination rates were the percentage of people in a population who have received at least one dose of a COVID-19 vaccine. Data were collected from an online database that updates global vaccination rates on a daily basis from a variety of official public sources (see [38] for details). Data were collected on August 30, and October 30, 2021. Because some regions do not update their vaccination rates on a daily basis, the most current vaccination rates available on each date were used.

### 2.8 Controlled factor criteria

In all studies, we tested for an association between spirituality and/or religiosity and COVID-19 vaccination rates, while controlling for age. Because there is likely an effect of age on vaccination rates, age was available for most regions, and there was an adequate sample size in all studies to include age, age was included in all analyses. However, all reported effects of spirituality and/or religiosity hold when age is not included in the analyses.

We then added additional controlled factors to the regression models depending on data availability and sample size restrictions. Our sample size guideline was at least 10 regions per predictor in our regression models. Including more predictors than this would increase the likelihood of overfitting the regression models and explaining error. All reported effects of spirituality and religiosity hold when these controlled factors are not included.

### 2.9 Region inclusion criteria

For Study 1, all countries that had spirituality and religiosity scores from a recently published study [11] and vaccination rates were included in the analyses, which consisted of 23 countries: Australia, Belgium, Brazil, Canada, Egypt, France, Germany, Iran, Israel, Italy, Mexico, Morocco, Netherlands, North Macedonia, Poland, Portugal, Romania, Sweden, Tunisia, Turkey, United Kingdom, United States, and Venezuela.

For Study 2, all regions that had data from the Wellcome Global Monitor [31] that contained religious data and had available vaccination rates were included, which consisted of 144 regions around the world.

For Study 3, all regions that had data from the Religious Landscape Study [30] and vaccination rates where included, which consisted of all 50 states within the USA in addition to the District of Columbia.

Across Studies, 195 unique regions from around the world were included.

## 3 Results

Descriptive statistics for the Studies are provided in Table 1. All regression analyses presented below were run while controlling for the demographic variable of median age of the region. We limited the number of predictors in Study 1 due to the relatively small sample size in order to ensure enough power to detect an effect and to avoid overfitting the regression models. However, all predictors, when available, were assessed together in the larger samples of Studies 2 and 3. There was no evidence of problematic multicollinearity among the predictors for any of the studies. Studies 1 and 2 variance inflation factor (VIF) < 2.3 and tolerance statistics > 0.41, and in Study 3 where spirituality and religiosity were compared together, VIF < 5.5 and tolerance statistics > 1.8. However, to account for any problematic multicollinearity issue in Study 3, we ran a series of regression analyses that varied when potentially overlapping predictors (i.e., religiosity and spirituality) were included: with spirituality (model 1), religiosity (model 2), and both (model 3).

**Table 1:** Descriptive Statistics for Studies 1–3.

	Study 1	Study 2	Study 3
*N*	23	144	51
Spirituality			
Mean (SD)	3.36 (0.53)		58.20 (6.36)
Min, Max	2.43, 4.74		43.00, 71.00
Religiosity			
Mean (SD)	33.11 (13.97)	89.64 (14.16)	52.65 (10.65)
Min, Max	15.24, 61.13	31.80, 100.00	32.00, 77.00
GDP			
Mean (SD)		15 081.90 (20 705.40)	65 458.82 (24 107.61)
Min, Max		274.00, 115 873.60	41 203.48, 213 939.63
Collectivism			
Mean (SD)		61.70 (22.02)	50.08 (11.34)
Min, Max		9.00, 94.00	31.00, 91.00
Vaccination rates (August)			
Mean (SD)	53.91 (20.72)	35.55 (26.53)	59.49 (8.80)
Min, Max	5.93, 84.95	0.00, 87.01	44.17, 76.08
Vaccination rates (October)			
Mean (SD)	62.69 (17.68)	43.14 (27.89)	64.32 (9.10)
Min, Max	16.06, 88.63	0.01, 96.62	48.95, 80.13

*Note*. Besides vaccination rates and GDP, scales used in each study varied. See Method section for details.

### 3.1 Study 1

**Fig. 1:**
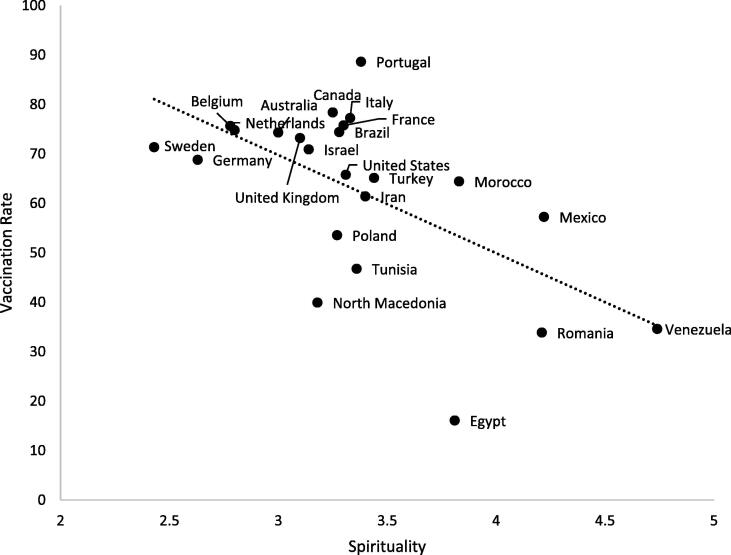
Correlation between spirituality and vaccination rates in October for Study 1, *r*(21) = −0.59, *P* <.01.

**Fig. 2:**
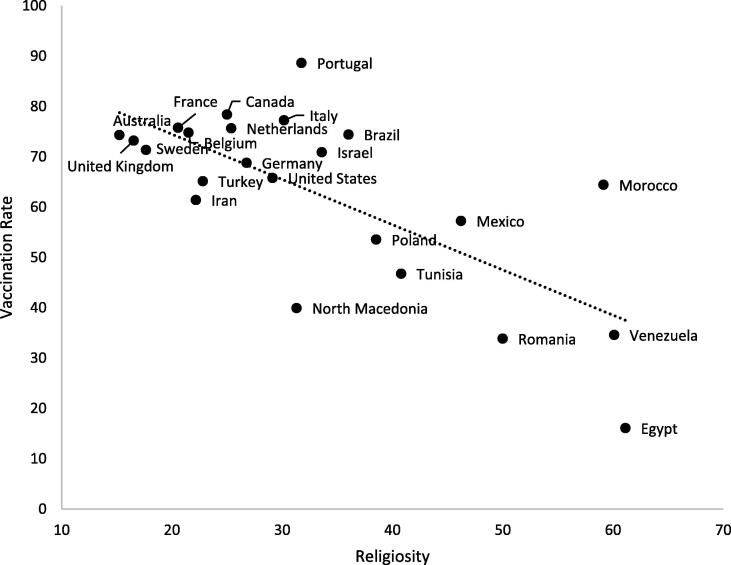
Correlation between religiosity and vaccination rates in October for Study 1, *r*(21) = −0.71, *P* <.001.

Linear regressions were run to predict vaccination rates from religiosity and spirituality (see Table 2 and Fig. 1, Fig. 2). If vaccination supply is a factor, then more recent vaccination rates should be more strongly influenced by spirituality and religiosity than older rates as supplies are more likely to be available later on, and results were consistent with this expectation. Religiosity was a marginally significant predictor during the earlier time period, β = −0.38, *p* =.058, but a significant predictor during the later time period, β = -0.61, *p* =.004. Similarly, spirituality was a marginally significant predictor during the earlier time period, β = −0.37, *p* =.060, but a significant predictor during the later time period, β = −0.44, *p* =.04.

**Table 2:** Regression Analyses of Spirituality and Religiosity on Vaccination Rates in Study 1.

	August					October				
	βs	R^2^	95% CI		*p*	βs	R^2^	95% CI		*p*
			*LL*	*UL*				*LL*	*UL*	
Spirituality	−.37^a^	0.45	−29.928	0.679	0.060	−0.44*	0.35	−28.830	−0.546	0.043
Spirituality (20%)	−0.44*	0.37	−29.663	−0.730	0.040	−0.55*	0.31	−27.708	−3.088	0.017
Religiosity	−.38^a^	0.45	−1.157	0.021	0.058	−0.61**	0.48	−1.256	−0.281	0.004
Religiosity (20%)	−0.33	0.31	−1.080	0.135	0.120	−0.57*	0.34	−1.135	−0.172	0.010

*Note.* βs are standardized. R^2^ are adjusted. CI = confidence interval; *LL* = lower limit; *UL* = upper limit. 20% values represent those with the at least 20% vaccination rates. All values are controlling for median age.

* *P* <.05. ** *P* <.01. ^a^*P* <.10.

As an alternative method to account for vaccine availability, the analyses were run again with a 20% threshold of vaccination rates. With the 20% threshold applied, religiosity was not a significant predictor during the earlier time period, β = −0.33, *p* >.05, but was a significant predictor during the later time period, β = −0.57, *p* =.01. Spirituality was a significant predictor both during the earlier time period, β = −0.44, *p* =.04, and later time period, β = −0.55., *p* =.02.

The results of the 20% threshold and earlier time-period methods produced similar results (i.e., statistically significant effects in the same predicted direction), and since assessing predictors on multiple dates does not reduce the sample size, the 20% threshold was dropped going forward.

Study 1 thus found support for our hypotheses. However, one limitation is that the sample size precluded us from controlling for other factors that might be influencing vaccination rates. Study 2 and 3 addressed this limitation by having samples of 144 and 51, respectively.

### 3.2 Study 2

Linear regressions with religiosity predicting vaccine rates during the earlier and later time periods were run (see Table 3), Religiosity was a significant predictor during the earlier time period, β = −0.49, *p* <.001, and later time period, β = −0.52, *p* <.001. Effects of religiosity controlling for GDP, collectivism, vaccine skepticism, previous inoculation history, and age held for the later time period, β = −0.21, *p* =.01, but not the earlier time period, β = −0.10, *p* =.20.

**Table 3:** Regression Analyses of Religiosity, GDP, Collectivism, Vaccine Skepticism, and Inoculation History in Study 2.

	August					October				
	βs	R^2^	95% CI		*p*	βs	R^2^	95% CI		*p*
			*LL*	*UL*				*LL*	*UL*	
Religiosity (alone)	−.12^a^	0.23	−0.468	0.035	0.091	−0.16*	0.56	−0.569	−0.049	0.020
Full Model		0.62					0.59			
Religiosity	−0.10		−0.418	0.089	0.202	−0.21*		−0.624	−0.081	0.012
GDP	0.42***		0.000	0.001	<0.001	0.33***		0.000	0.001	<0.001
Collectivism	0.09		−0.091	0.295	0.298	0.14		−0.047	0.366	0.129
Skepticism	−0.33***		−41.952	−15.141	<0.001	−0.36***		−45.978	−17.253	<0.001
Inoculation	0.02		−89.412	131.589	0.706	0.01		−113.906	122.881	0.940

*Note.* βs are standardized. R^2^ are adjusted. CI = confidence interval; *LL* = lower limit; *UL* = upper limit. All values are controlling for median age.

* *P* <.05. *** *P* <.001. ^a^*P* <.10.

### 3.3 Study 3

**Fig. 3:**
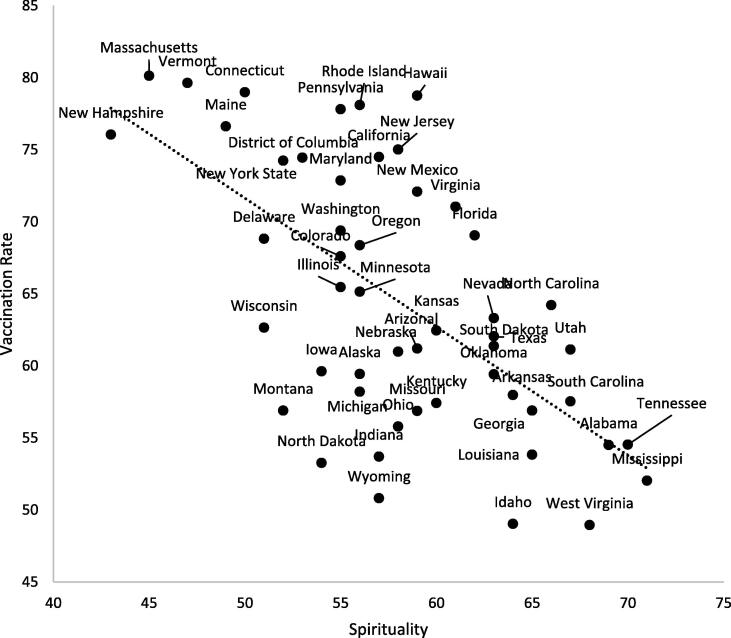
Correlation between spirituality and vaccination rates in October for Study 3, *r*(49) = −0.62, *P* <.001.

**Fig. 4:**
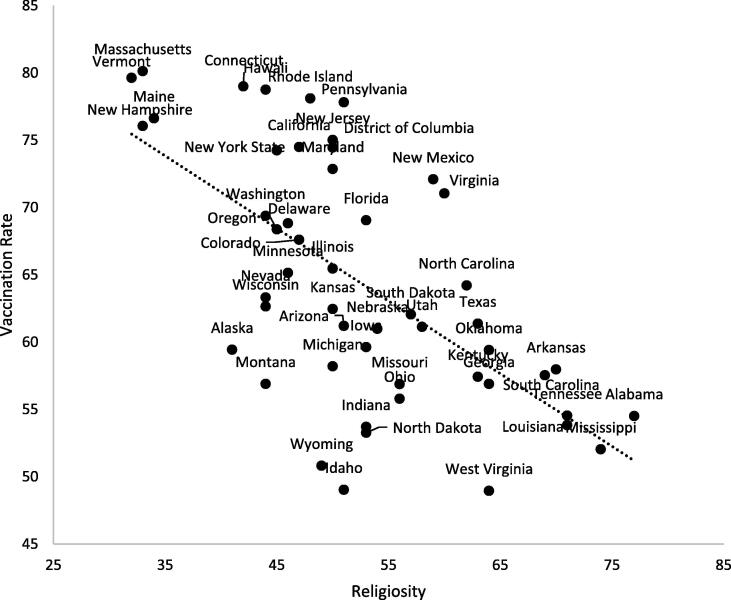
Correlation between religiosity and vaccination rates in October for Study 3, *r*(49) = −0.63, *P* <.001.

Linear regressions with religiosity predicting vaccine rates during the earlier and later time periods were run (see Table 4 and Fig. 3, Fig. 4), Religiosity was a significant predictor both during the earlier time period, β = −0.59, *p* <.001, and the later time period, β = −0.58, *p* <.001, as was spirituality, β = −0.59 and −0.58, respectively, *p*s < 0.001. Effects of religiosity controlling for GDP, collectivism, and age held for both time periods, β = −0.51 and −0.49, respectively, *p*s < 0.001, as did the effects of spirituality for the earlier and later time period, β = −0.51, *p* =.002, and β = −0.47, *p* =.004. However, with both religiosity and spirituality included in the model, only religiosity was significant during the most recent time period (see Table 4 for details).

**Table 4:** Regression Analyses of Spirituality, Religiosity, GDP, and Collectivism on Vaccination Rates in Study 3.

	August					October				
	βs	R^2^	95% CI		*p*	βs	R^2^	95% CI		*p*
			*LL*	*UL*				*LL*	*UL*	
Spirituality (alone)	−0.59***	0.40	−1.146	−0.497	<0.001	−0.58***	0.38	−1.167	−0.485	<0.001
Religiosity (alone)	−0.59***	0.41	−0.679	−0.301	<0.001	−0.58***	0.40	−0.696	−0.301	<0.001
Model 1		0.57					0.57			
Spirituality	−0.51**		−1.127	−0.277	0.002	−0.47**		−1.099	−0.227	0.004
GDP	0.31*		0.000	0.000	0.025	0.34*		0.000	0.000	0.013
Collectivism	0.35**		0.103	0.434	0.002	0.35**		0.113	0.453	0.002
Model 2		0.59					0.60			
Religiosity	−0.51***		−0.635	−0.193	<0.001	−0.49***		−0.637	−0.189	<0.001
GDP	0.32*		0.000	0.000	0.012	0.33**		0.000	0.000	0.007
Collectivism	0.33**		0.103	0.416	0.002	0.35***		0.121	0.438	<0.001
Model 3		0.59					0.60			
Spirituality	−0.22		−0.895	0.301	0.322	−0.15		−0.828	0.392	0.475
Religiosity	−.37^a^		−0.619	0.020	0.065	−0.39*		−0.655	−0.004	0.048
GDP	0.27*		0.000	0.000	0.042	0.30*		0.000	0.000	0.024
Collectivism	0.36**		0.118	0.441	0.001	0.37***		0.130	0.459	<0.001

*Note.* βs are standardized. R^2^ are adjusted. CI = confidence interval; *LL* = lower limit; *UL* = upper limit. All values are controlling for median age.

* *P* <.05. ** *P* <.01. *** *P* <.001. ^a^*P* <.10.

## 4 Discussion

The purpose of the current research was to assess whether spirituality and religiosity—measured on the region-level (i.e., countries or states)—are associated with ongoing COVID-19 vaccination rates. The results support the hypotheses: in Study 1, both spirituality and religiosity were individually associated with vaccination rates, such that the higher the spirituality/religiosity of the region, the lower the vaccination rates. These effects held when applying two methods to account for vaccine supply issues: a 20% vaccine threshold and when assessing vaccination rates at different time periods. Results were similar for both spirituality and religiosity. The βs and R^2^ values suggest that religiosity is generally slightly more strongly associated with vaccination rates than spirituality.

We built on this initial finding in Study 2, with a larger global sample. Once again, religiosity was significantly negatively associated with vaccination rates. The effect held when controlling for general vaccine skepticism, previous inoculation history, collectivism, GDP, and region age. Contrary to prior research [26], previous inoculation history was not reliably associated with vaccination rates at any time period, which suggests that the current pandemic might be influencing people of a given region differently than it had before. One possibility for this difference is that the novelty of COVID-19 vaccines might be increasing stress and anxiety, which might not have been as large of a factor with previous vaccinations. It is also possible that being vaccinated during a pandemic versus “regular” vaccinations that have a longer vaccination history, has less unknowns associated with the vaccines themselves. However, general vaccine skepticism was associated with COVID-19 vaccination rates at both time periods, which suggests that vaccine skepticism, rather than previous vaccination behavior, is a factor influencing COVID-19 vaccination rates. Taken together with the religiosity effect, these results suggest that religiosity at the region-level is associated with real-world behavior in the form of vaccination rates beyond the general level of vaccine skepticism of a region, and that the level of previous inoculations is, in this case, poorly associated with COVID-19 vaccination rates. The βs for religiosity were smaller than in Study 2, but still contributing to the models.

We further built on these findings in Study 3, but this time using data from all USA states and the District of Columbia, which showed similar patterns. Individually, spirituality and religiosity again were significantly associated with COVID-19 vaccination rates. The effects of spirituality and religiosity held controlling for collectivism, GDP, and region age. However, when religiosity and spirituality were included in the same model, only religiosity was associated with vaccination rates. This finding suggests, contrary to previous research [11], that religiosity and not spirituality is more strongly associated with vaccination rates. It is not clear why this difference emerged here, but given the current difficulty in measuring spirituality at this level of analysis (discussed further below) and that the findings differ from those on the individual-level, these results should be interpreted with caution until they are replicated. For now, previous research suggests spirituality is the stronger contributor at the individual-level [11], but possibly not be on the region-level. Future research should attempt to replicate these findings more directly.

Because Study 3 consisted of different regions within a single country (i.e., USA), various confounding cultural factors that are present in the global samples of Studies 1 and 2 are less likely to be present in the USA sample of Study 3. For example, although there are cultural differences between each U.S. state, the states are likely more similar to each other than they are to countries such as Tajikistan, Greece, Jordan, or Japan [40]. The inclusion of Study 3 thus lessens—though not eliminates—concerns of confounding cultural variables. This is an important point, as cross-cultural comparisons can potentially be influenced by many cultural differences, but by looking within a single country, many of these differences are controlled for.

An important contribution of the current research is that it assessed real-world behavior through vaccination rates. Previous work on vaccination skepticism has largely focused on attitudes assessed through surveys. The results from Study 2 suggest that religiosity explains additional variance in vaccination rates than general attitudes about vaccinations. However, the behavioral data of the current research converges with research assessing attitudes of spirituality and religiosity to suggest that spirituality and religiosity individually predict vaccination behavior. This converging evidence increases confidence in spirituality and religiosity in particular being reliable predictors.

It is worth pointing out that the results are correlational in nature. Spirituality and religiosity are, individually, associated with COVID-19 vaccination rates. However, despite the correlational nature, it makes logical sense that the relationship is causal in that spirituality and religiosity are leading to lower vaccination rates. First, although religiosity can change throughout the lifespan, it tends to take years to do so [41], so it seems unlikely that skepticism about or refusal of COVID-19 vaccination is causing people to become less religious or spiritual. It makes much more sense that religious and spiritual beliefs are causing less COVID-19 vaccinations. In addition, there could be third variables that are leading to this association. Political conservativism, which we expand on later, might be one such third variable. Although it is possible that the link between religiosity/spirituality and COVID-19 vaccinations is causal, such an interpretation is premature and should be done cautiously, as causality has not been established. Future research is necessary to establish the causal link and test for additional third variables that might contribute to this association.

Although the current work did not assess why spirituality and religiosity are associated with COVID-19 vaccination rates, we can speculate beyond what was discussed in the introduction. The results emerged globally, so this suggests that it is not a particular religious or spiritual belief that is driving the effect. In fact, spiritual beliefs tend to be quite individualized in nature, so spiritual beliefs are likely to be somewhat unique to each individual [9]. Rather, it seems that certain aspects of these beliefs in general are leading to lower COVID-19 vaccination rates (e.g., misalignment between the strong reliance on intuition and subjective experience that generally characterized these beliefs and the counterintuitive nature of vaccination, perceptions of vaccination as unnatural that clash with a strong preference for naturalness [7], [9], [11]). This is speculation, as the data suggests an association, not a causal link. Ironically, few religions actually ban vaccinations [42], yet historically, religion has been used as a reason to avoid vaccinations, which can be dated back to the very first vaccine in 1796 [43]. The dichotomy between religions not explicitly banning vaccines and religion being used as a reason to be exempt from them requires further research, but it appears that it is a broad effect that is related to many spiritual and religious doctrines.

A related point relates to what might be done to help promote COVID-19 vaccinations given their health benefits. One potential avenue might be to tailor messages towards those more religious and spiritual. Indeed, when someone advocating for vaccinations shares a common religion, religious individuals are more likely to support vaccines [44]. Some religious leaders have explicitly encouraged COVID-19 vaccinations, such as the highest clergy member of the Greek Orthodox Church in America: “Today the Holy Eparchial Synod declared that there is no religious exemption from any vaccine, including the COVID19 vaccine” [45]. Though clearly other religious leaders have not made the same type of statements and often encourage vaccine hesitancy, which might have the opposite effect of promoting such hesitancy.

Importantly, the ecological fallacy suggests that data collected on one level of analysis should not be blindly applied to another. The current work was conducted on the region-level (i.e., countries and states), so applying the effects to individuals might not be appropriate. Nonetheless, our results converge somewhat with those previously done at the individual-level to suggest that religion and spirituality are associated with COVID-19 vaccination rates at both levels of analysis, so efforts at the individual-level (e.g., tailoring messages towards individual beliefs) might similarly lead to region-level effects. Importantly, this is an area for future research to assess, as this is currently speculation.

There are several limitations worth mentioning. First, assessing vaccination rates is difficult at the current stage because they are likely influenced by a variety of ongoing factors, such as supply issues and complicated vaccine roll-outs. Although efforts were made to control for supply issues by including a threshold of at least 20% vaccination rates (Study 1), including GDP as a predictor (Studies 2 and 3), and comparing earlier vaccine rates to more recent ones (Studies 1–3), confounding factors might still be present. Some of these factors are less likely to be present in Study 3, which relied on a single country that had national distribution of COVID-19 vaccinations, but disparities might still be present in how vaccines were administered. Nonetheless, the current nature of the data limits what conclusions can be drawn.

In addition, because vaccination programs are still ongoing, it is not clear how well spirituality and religiosity will predict COVID-19 vaccination rates once the various global vaccination drives have essentially finished. The data generally suggest that spirituality and religiosity are good predictors later on in the pandemic on a global level, but once anxieties have decreased and social restrictions eased, attitudes and behavior might change. However, within the U.S. sample, spirituality and religiosity were not always stronger predictors later on, which suggests that the time period where spirituality and religiosity have the greatest impact might be dependent on the context. Ongoing assessments of how spirituality and religiosity play a role in vaccinations are needed.

Lastly, there are limits to how spirituality was measured. Spirituality was most directly measured in Study 1, while the spirituality measure in Study 3 was operationalized slightly differently by assessing spiritual experiences. Despite these differences, results converged, which suggests spirituality is a somewhat robust predictor. Global spirituality data is more difficult to come by than religiosity data, yet it appears to be an important and underutilized predictor. There is a need for a global spirituality index—similar to efforts to measure religiosity globally (e.g., [30]). Taken together, spirituality at the region-level (whether the population considers themselves spiritual or has spiritual experiences) predicts ongoing vaccination rates.

Future research would benefit from assessing the possibility of there being alternative explanations. Political conservativism might be an important factor, particularly in the USA—though perhaps not more globally (11). Previous research on individual-level predictors of vaccine acceptance, however, did not find effects of conservatism. For example, controlling for conservatism in multiple regression analyses did not alter the effects that were observed: specifically, of religiosity (10), spirituality (9,11), and science literacy (9–11). Even when specifically gauging COVID-19 related vaccine acceptance, the role of political ideology has been shown to be minimal (46). Thus, while on the individual level conservatism does not seem to be a predictor of vaccine acceptance (18), it could still be a more prominent predictor on the group level. Therefore, we believe its inclusion would be a worthwhile area for future research.

## 5 Conclusions

Individually, both spirituality and religiosity at the region-level are associated with ongoing COVID-19 vaccination rates, and they are not due to differences in GDP, collectivism, general vaccine skepticism, inoculation history, or age of the population. These results are consistent with and extent research done on the individual-level [11], [19], and suggest that real-world behavior (i.e., COVID-19 vaccinations) can be predicted by the relative spirituality and/or religiosity of a region.

## Data availability

All data used in the analyses are freely availability online through the original sources. See predictor and outcome variables sections above for references.

## Declaration of Competing Interest

The authors declare that they have no known competing financial interests or personal relationships that could have appeared to influence the work reported in this paper.

## Data Availability

Data will be made available on request.
